# Supplementary material for: Gene Expression Signature Predictive of Neuroendocrine Transformation in Prostate Adenocarcinoma
Source: Int J Mol Sci. 2020 Feb 6;21(3):1078. doi: 10.3390/ijms21031078 (PMC7037893; doi:10.3390/ijms21031078)
Supplement: Supplementary file 1 [file ijms-21-01078-s001.zip › ijms-690854-supplementary-final/Supplementary_material/Supplementary Figures.docx]

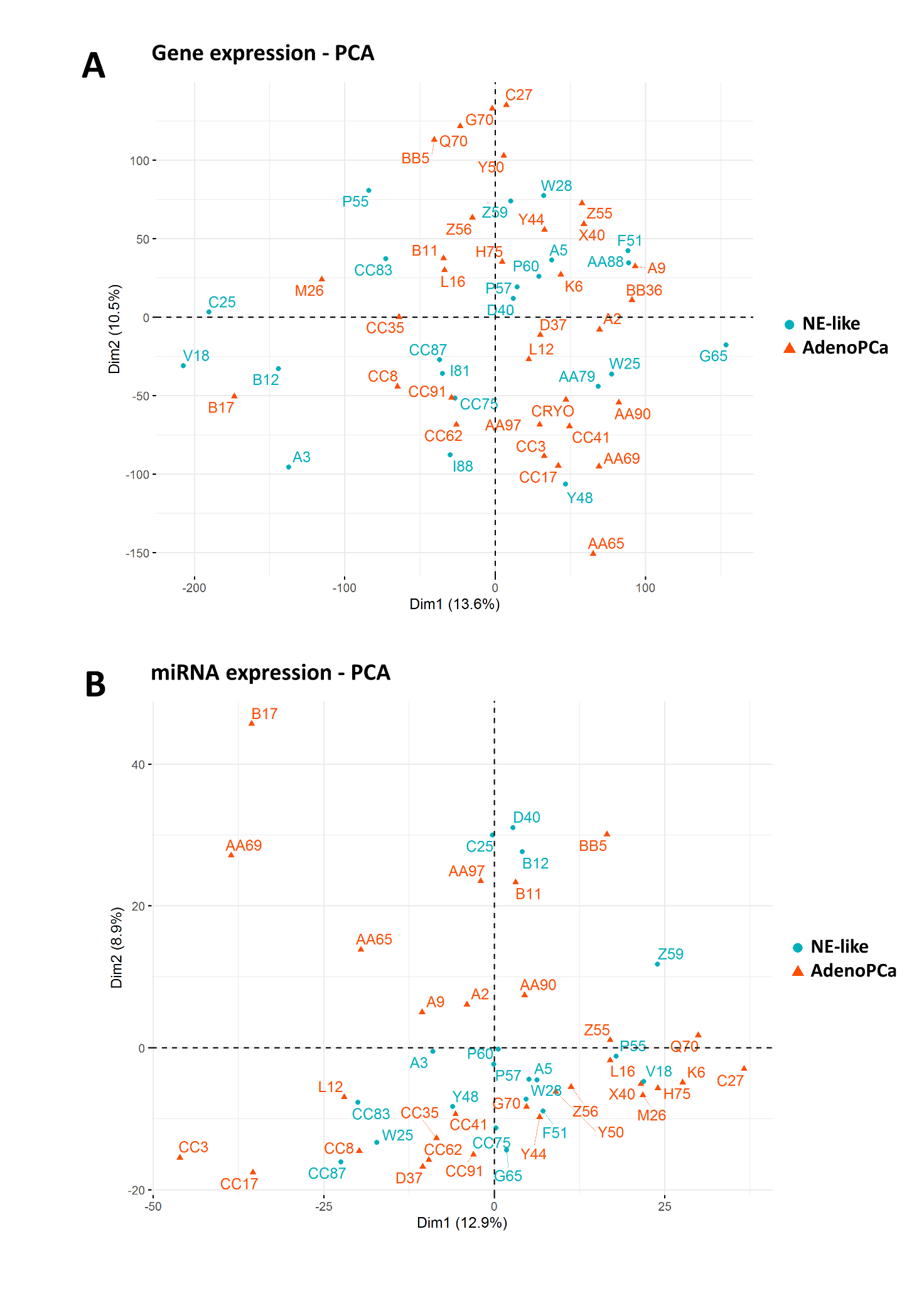


**Supplementary Figure 1. Data reduction analysis on gene and miRNA expression profiles of our cohort of prostate tumor samples. Principal Component Analysis (PCA) was performed on coding and non-coding RNA (A) and miRNA (B) expression profiles from NE-like (blue dots) and AdenoPCa samples (red triangles).**


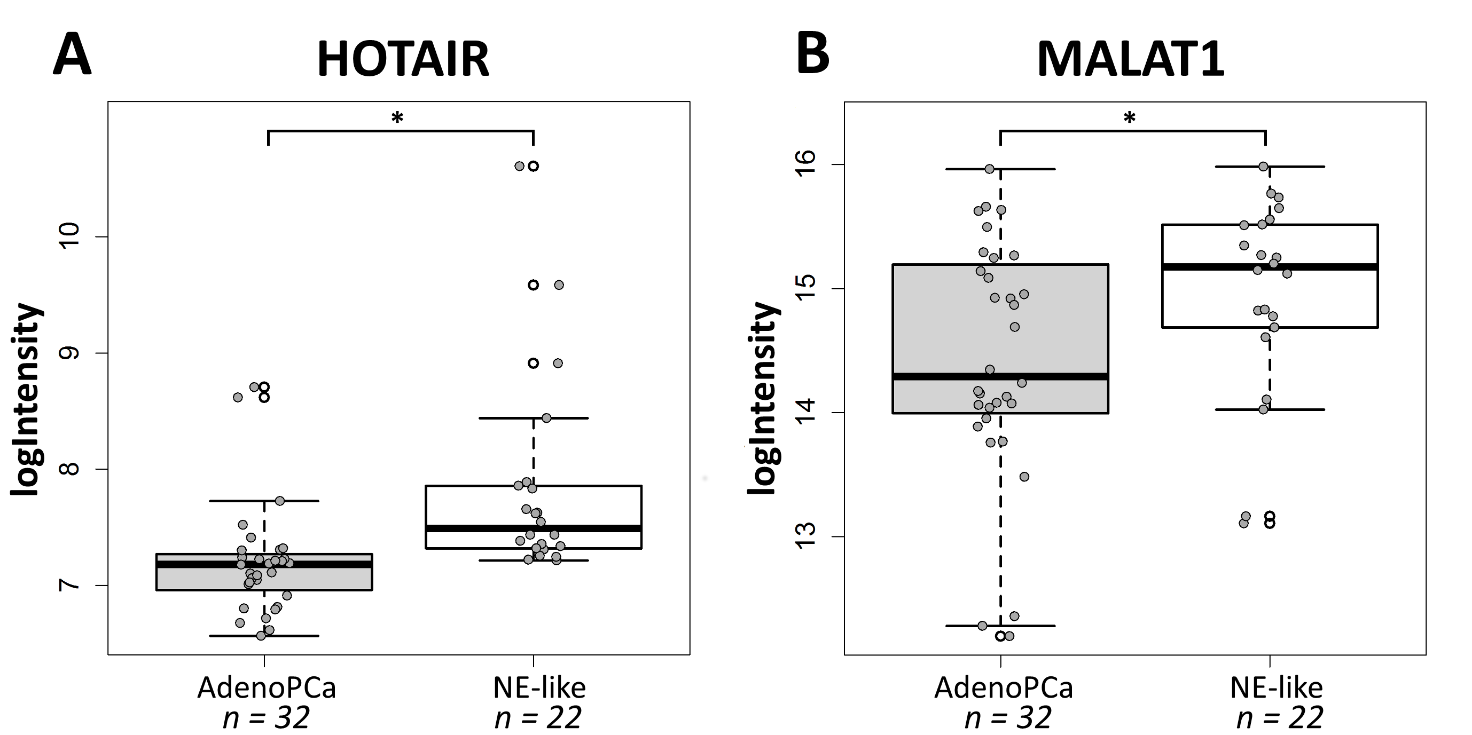


**Supplementary Figure 2. Boxplot representation of logIntensity values for Hotair (A) and Malat1 (B). Both transcripts were up-regulated in NE-like (n = 22) vs AdenoPCa samples (n = 32) with logFC = 0.63 and logFC = 0.53 and pvalue <** **0.05, respectively. Significant differences (p < 0.05) are marked with an asterisk.**


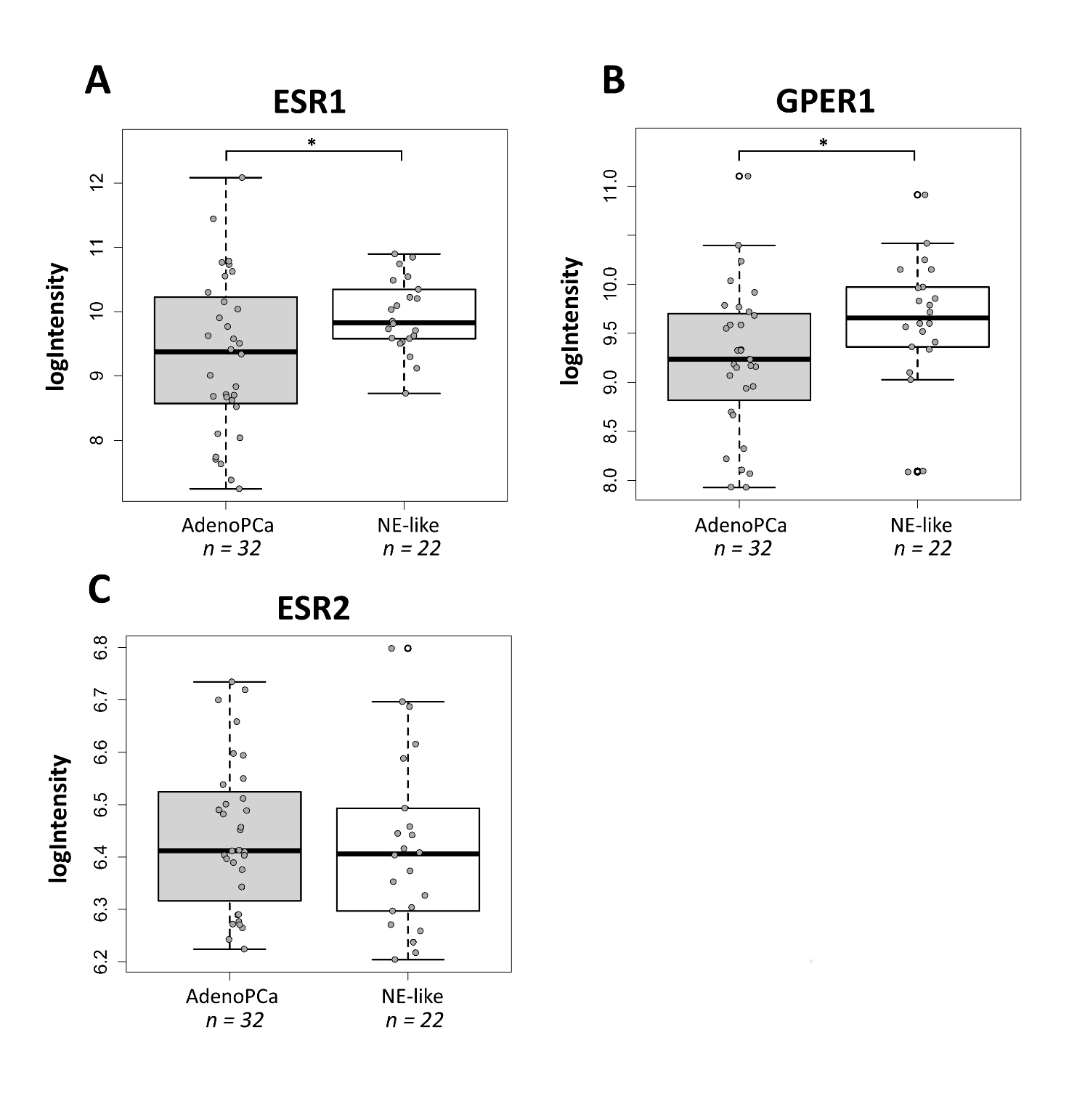


**Supplementary Figure 3. Boxplot representation of logIntensity values for ESR1 (A), GPER1 (B) and ESR2 (C). ESR1 and GPER1 were up-regulated in NE-like vs AdenoPCa samples with logFC = 0.6, p-value = 0.03 and and logFC = 0.4, p-value =** **0.047, respectively, while ESR2 had similar expression in the two groups. Significant differences (p < 0.05) are marked with an asterisk. Median expression of ESR2 was lower if compared to ESR1 and GPER1 expression.**


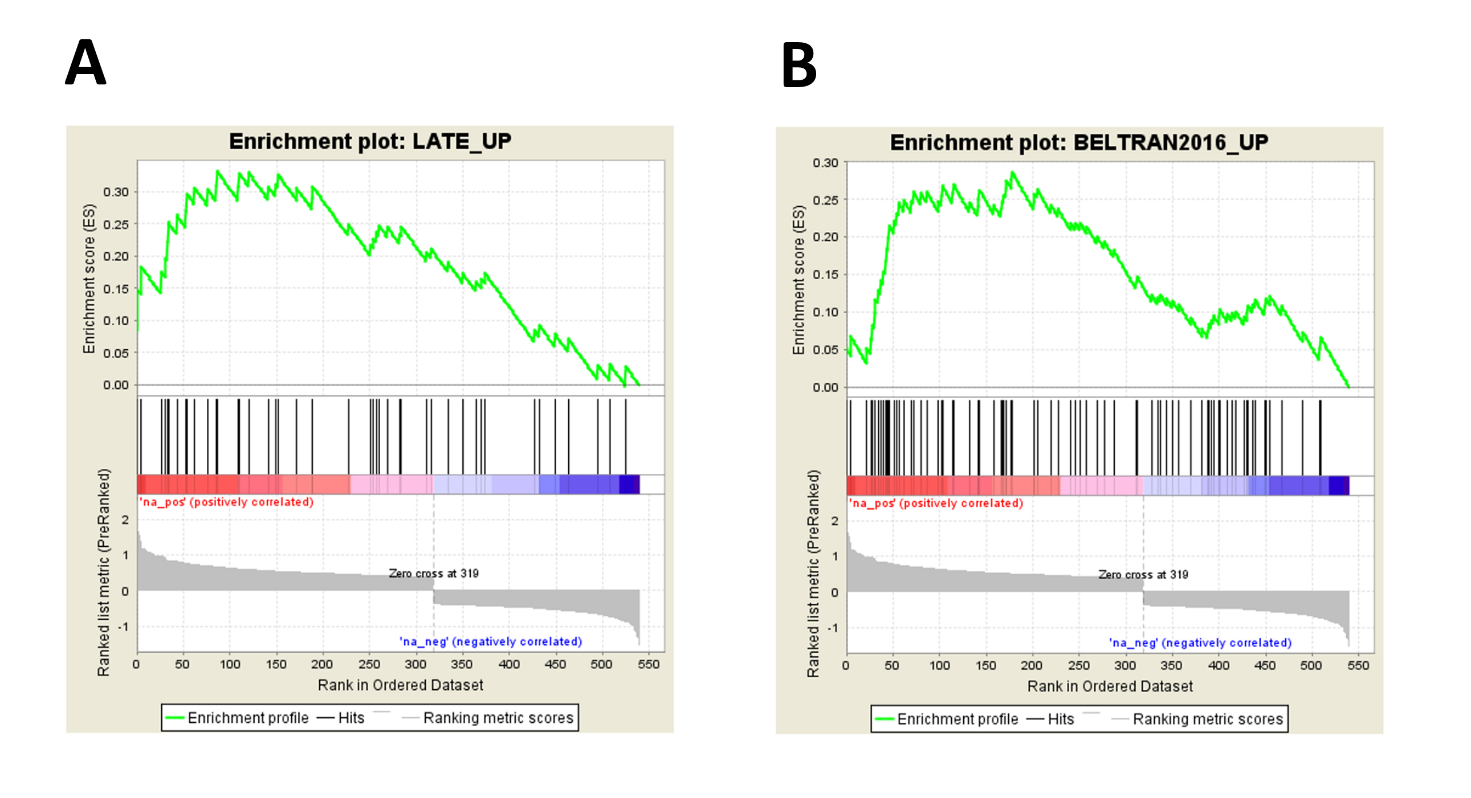


**Supplementary Figure 4. Gene set enrichment analysis (GSEA) performed in pre-ranked mode using differentially expressed genes in NE-like vs AdenoPCa samples. Genes more expressed in NE-like tumors (on the left) are associated with up-regulated genes in late neuroendocrine signature from Mauri et al. [16] (Nominal p-value = 0.008, FDR q-value = 0.028, NES = 1.81) and with upregulated genes in CRPC-NE tumors from Beltran et al. [4] (Nominal p-value = 0.004, FDR q-value = 0.006, NES = 1.85) in a statistical significant manner.**
