# Supplementary material for: Gene Expression Signature Predictive of Neuroendocrine Transformation in Prostate Adenocarcinoma
Source: Int J Mol Sci. 2020 Feb 6;21(3):1078. doi: 10.3390/ijms21031078 (PMC7037893; doi:10.3390/ijms21031078)
Supplement: Supplementary file 1 [file ijms-21-01078-s001.zip › ijms-690854-supplementary-final/Supplementary_material/Supplementary Table 2.docx]

**Supplementary Table 2**. List of common and specific ESR1/AR transcriptional targets, log2 Fold Change values and the mechanism of regulation, if known.

| From | To | logFC in NE-like vs AdenoPCa | Mechanism |
| --- | --- | --- | --- |
| AR; ESR1 | IGF1 | -0.84 | Activation; Activation |
| AR; ESR1 | LDHA | -0.64 | Activation; Activation |
| AR; ESR1 | RUNX2 | 0.39 | Inhibition in PCa; Activation in PCa |
| AR; ESR1 | GPER1 | 0.39 | Unspecified; Inhibition |
| AR; ESR1 | KRT7 | 0.47 | Activation; Inhibition |
| AR; ESR1 | RERG | 0.49 | Activation; Activation |
| AR; ESR1 | UGT2B15 | 0.55 | Inhibition in PCa; Activation |
| AR; ESR1 | HOTAIR | 0.63 | Inhibition in PCa; mainly activated |
| AR; ESR1 | DDC | 0.96 | Unspecified; Unspecified |
| AR; ESR1 | AGR3 | 1.03 | Activation; Activation |
| AR; ESR1 | S100P | 1.06 | Activation; Activated in PCa |
| AR; ESR1 | AGR2 | 1.14 | Activation; Activation |
| AR; ESR1 | PSCA | 1.55 | Activation; Inhibition |
| ESR1 | H19 | -1.15 | Activation |
| ESR1 | HAPLN1 | -0.80 | Unspecified |
| ESR1 | SERPING1 | -0.69 | Inhibition |
| ESR1 | OLFML3 | -0.64 | Activation |
| ESR1 | PTGS1 | -0.59 | Activation |
| ESR1 | KCNMA1 | -0.57 | Activation |
| ESR1 | AR | -0.55 | Unspecified |
| ESR1 | eIF2B3 | -0.53 | Activation |
| ESR1 | PEMT | -0.50 | Unspecified |
| ESR1 | NAV3 | -0.46 | Unspecified |
| ESR1 | RIMS4 | -0.43 | Activation |
| ESR1 | mTOR | -0.42 | Activation |
| ESR1 | FMO3 | -0.39 | Inhibition |
| ESR1 | PSMD14 | -0.38 | Unspecified |
| ESR1 | EGLN3 | 0.38 | Activation |
| ESR1 | Rel | 0.40 | Unspecified |
| ESR1 | Clmn | 0.41 | Inhibition |
| ESR1 | NGEF | 0.42 | Unspecified |
| ESR1 | PLEKHF2 | 0.42 | Inhibition |
| ESR1 | PREX1 | 0.42 | Inhibition |
| ESR1 | ATP11A | 0.44 | Activation |
| ESR1 | MALL | 0.44 | Inhibition |
| ESR1 | S100A10 | 0.46 | Unspecified |
| ESR1 | Bcl3 | 0.46 | Inhibition |
| ESR1 | CGN | 0.47 | Inhibition |
| ESR1 | GJB2 | 0.48 | Activation |
| ESR1 | KLK1 | 0.50 | Activation |
| ESR1 | SLC12A2 | 0.50 | Inhibition |
| ESR1 | PLEKHG7 | 0.51 | Unspecified |
| ESR1 | Myb | 0.52 | Activation |
| ESR1 | MLPH | 0.56 | Inhibition |
| ESR1 | BATF | 0.58 | Inhibition |
| ESR1 | CGA | 0.63 | Inhibition |
| ESR1 | BTG2 | 0.67 | Inhibition |
| ESR1 | KRT4 | 0.72 | Inhibition |
| ESR1 | TFF1 | 0.96 | Activation in PCa |
| ESR1 | TFCP2L1 | 0.97 | Unspecified |
| ESR1 | REG4 | 1.07 | Unspecified |
| AR | CXCL13 | -1.27 | Activation in PCa |
| AR | NPR3 | -1.04 | Unspecified in PCa |
| AR | DCN | -0.96 | Unspecified |
| AR | VCAN | -0.84 | Activation in PCa |
| AR | CA3 | -0.83 | Unspecified in PCa |
| AR | GNMT | -0.81 | Activation in PCa |
| AR | PTN | -0.76 | Unspecified in PCa |
| AR | FKBP5 | -0.59 | Activation in PCa |
| AR | G0S2 | -0.59 | Unspecified in PCa |
| AR | SYNGR1 | -0.59 | Unknown in PCa |
| AR | CaMKK2 | -0.57 | Activation in PCa |
| AR | MT1X | -0.54 | Activation in PCa |
| AR | ADH1A | -0.53 | Unknown |
| AR | EMP3 | -0.47 | Unspecified in PCa |
| AR | ARMC12 | -0.38 | Activation in PCa |
| AR | CHN2 | 0.38 | Unspecified in PCa |
| AR | NR6A1 | 0.40 | Inhibition in PCa |
| AR | WNK2 | 0.41 | Unspecified |
| AR | PDGFRL | 0.43 | Inhibition in PCa |
| AR | GULP1 | 0.43 | Unspecified in PCa |
| AR | DMD | 0.47 | Unspecified in PCa |
| AR | AMIGO2 | 0.51 | Inhibition in PCa |
| AR | DUSP4 | 0.55 | Unspecified in PCa |
| AR | UBE2E3 | 0.57 | Unspecified in PCa |
| AR | TSPAN8 | 0.59 | Unspecified in PCa |
| AR | CLDN4 | 0.59 | Unspecified in PCa |
| AR | AMD1 | 0.59 | Activation in PCa |
| AR | B3GNT5 | 0.60 | Unspecified in PCa |
| AR | STEAP4 | 0.64 | Activation in PCa |
| AR | CDK19 | 0.65 | Unspecified in PCa |
| AR | POF1B | 0.68 | Unspecified in PCa |
| AR | CLDN3 | 0.70 | Activation in PCa |
| AR | GLDC | 0.71 | Unknown |
| AR | KLF5 | 0.71 | Activation in PCa |
| AR | GSTA2 | 0.75 | Unspecified in PCa |
| AR | SCGB2A1 | 0.82 | Activation in PCa |
| AR | ZNF385B | 0.94 | Unspecified in PCa |
| AR | FOLH1 | 0.96 | Activation in PCa |
| AR | NEK2 | 1.06 | Unspecified in PCa |
| AR | KLK11 | 1.11 | Activation |
| AR | TGM4 | 1.18 | Activation |
