# Supplementary material for: Gene Expression Signature Predictive of Neuroendocrine Transformation in Prostate Adenocarcinoma
Source: Int J Mol Sci. 2020 Feb 6;21(3):1078. doi: 10.3390/ijms21031078 (PMC7037893; doi:10.3390/ijms21031078)
Supplement: Supplementary file 1 [file ijms-21-01078-s001.zip › ijms-690854-supplementary-final/Supplementary_material/Supplementary Table 3.docx]

**Supplementary Table 3.** Functional enrichment analysis of genes up-regulated in an OSC model treated with estradiol. Terms related to neuroendocrine processes and pathways with a p-value < 0.05 were reported.

| **OSC11 treated with Estradiol** | | |
| --- | --- | --- |
| **Term - BP_ALL_UP** | **Count** | **P-Value** |
| GO:0046903~secretion | 54 | 0.001065 |
| GO:0050801~ion homeostasis | 35 | 0.007381 |
| GO:2000609~regulation of thyroid hormone generation | 3 | 0.009163 |
| GO:0010817~regulation of hormone levels | 24 | 0.025922 |
| GO:0030182~neuron differentiation | 51 | 0.03184 |
| **Term – CC_ALL_UP** | **Count** | **P-Value** |
| GO:0031982~vesicle | 198 | 2.8E-12 |
| GO:0099503~secretory vesicle | 31 | 7.45E-03 |
| GO:0030133~transport vesicle | 25 | 4.87E-02 |
| **Term - Maps_UP** | **Count** | **P-Value** |
| [Neurophysiological process_Dynein-dynactin motor complex in axonal transport in neurons](http://portal.genego.com/cgi/imagemap.cgi?id=6599) | 54 | 1.27E-02 |
| [Neuroprotective action of lithium](http://portal.genego.com/cgi/imagemap.cgi?id=5634) | 63 | 2.34E-02 |
| [Neurophysiological process_Constitutive and regulated NMDA receptor trafficking](http://portal.genego.com/cgi/imagemap.cgi?id=6561) | 65 | 2.64E-02 |
