# Supplementary material for: Gene Expression Signature Predictive of Neuroendocrine Transformation in Prostate Adenocarcinoma
Source: Int J Mol Sci. 2020 Feb 6;21(3):1078. doi: 10.3390/ijms21031078 (PMC7037893; doi:10.3390/ijms21031078)
Supplement: Supplementary file 1 [file ijms-21-01078-s001.zip › ijms-690854-supplementary-final/Supplementary_material/Supplementary Table 4.docx]

**Supplementary Table 4.** Functional enrichment analysis of genes up-regulated in VCaP cells overexpressing ESR1. Terms related to neuroendocrine processes and pathways with a p-value < 0.05 were reported.

| **GSE43988 – VCaP cells overexpressing ESR1** | | | |
| --- | --- | --- | --- |
| **Term - BP_ALL_UP** | **Count** | **%** | **P-Value** |
| GO:0048857~neural nucleus development | 8 | 1.684211 | 0.001304 |
| GO:0010817~regulation of hormone levels | 23 | 4.842105 | 0.002182 |
| GO:0046903~secretion | 41 | 8.631579 | 0.004326 |
| GO:0042391~regulation of membrane potential | 18 | 3.789474 | 0.00552 |
| GO:0051937~catecholamine transport | 6 | 1.263158 | 0.010248 |
| GO:0051047~positive regulation of secretion | 17 | 3.578947 | 0.010602 |
| GO:0032940~secretion by cell | 35 | 7.368421 | 0.013476 |
| GO:0060052~neurofilament cytoskeleton organization | 3 | 0.631579 | 0.014206 |
| GO:0009914~hormone transport | 15 | 3.157895 | 0.014466 |
| GO:0046879~hormone secretion | 14 | 2.947368 | 0.023743 |
| GO:0042445~hormone metabolic process | 10 | 2.105263 | 0.028435 |
| GO:0006812~cation transport | 33 | 6.947368 | 0.038404 |
| GO:0046883~regulation of hormone secretion | 12 | 2.526316 | 0.039598 |
| GO:0034754~cellular hormone metabolic process | 7 | 1.473684 | 0.041478 |
| GO:0001505~regulation of neurotransmitter levels | 10 | 2.105263 | 0.044932 |
| **Term_MF_ALL_UP** | **Count** | **%** | **P-Value** |
| GO:0043167~ion binding | 133 | 28 | 5.95E-04 |
| GO:0005497~androgen binding | 3 | 0.631579 | 0.003192 |
| GO:0030284~estrogen receptor activity | 3 | 0.631579 | 0.005238 |
| GO:0042562~hormone binding | 6 | 1.263158 | 0.017043 |
| GO:0015075~ion transmembrane transporter activity | 30 | 6.315789 | 0.017643 |
| GO:0008324~cation transmembrane transporter activity | 24 | 5.052632 | 0.01985 |
| GO:0005267~potassium channel activity | 8 | 1.684211 | 0.023027 |
| **Term_CC_ALL_UP** | **Count** | **%** | **P-Value** |
| GO:0031982~vesicle | 113 | 23.78947 | 0.001504 |
| GO:0099503~secretory vesicle | 21 | 4.421053 | 0.006108 |
| GO:0097458~neuron part | 46 | 9.684211 | 0.006581 |
| GO:0030141~secretory granule | 17 | 3.578947 | 0.009281 |
| GO:0005883~neurofilament | 3 | 0.631579 | 0.013533 |
| GO:0044456~synapse part | 23 | 4.842105 | 0.024976 |
| GO:0045202~synapse | 27 | 5.684211 | 0.027872 |
| GO:0030424~axon | 17 | 3.578947 | 0.033356 |
| GO:0043025~neuronal cell body | 17 | 3.578947 | 0.034251 |
| GO:0031594~neuromuscular junction | 5 | 1.052632 | 0.037752 |
| GO:0043005~neuron projection | 32 | 6.736842 | 0.039689 |
| GO:0043209~myelin sheath | 9 | 1.894737 | 0.048808 |
