# Supplementary material for: Gene Expression Signature Predictive of Neuroendocrine Transformation in Prostate Adenocarcinoma
Source: Int J Mol Sci. 2020 Feb 6;21(3):1078. doi: 10.3390/ijms21031078 (PMC7037893; doi:10.3390/ijms21031078)
Supplement: Supplementary file 1 [file ijms-21-01078-s001.zip › ijms-690854-supplementary-final/Supplementary_material/Supplementary Table 5.docx]

**Supplementary Table 5.** Functional enrichment analysis of genes up-regulated in LNCaP cells treated with estradiol. Terms related to neuroendocrine processes and pathways with a p-value < 0.05 were reported.

| **GSE37531 – LNCaP cells treated with estradiol** | | |
| --- | --- | --- |
| **Term_KEGG_UP** | **Count** | **P-Value** |
| h_agrPathway:Agrin in Postsynaptic Differentiation | 4 | 0.046317 |
| **Term - BP_ALL_UP** | **Count** | **P-Value** |
| GO:0044060~regulation of endocrine process | 6 | 0.00205 |
| GO:0050886~endocrine process | 8 | 0.0023 |
| GO:0010817~regulation of hormone levels | 19 | 0.015967 |
| GO:0046883~regulation of hormone secretion | 12 | 0.022742 |
| GO:2000831~regulation of steroid hormone secretion | 3 | 0.046028 |
| **Term - Networks_UP** | **Count** | **P-Value** |
| [Signal transduction_Neuropeptide signaling pathways](http://portal.genego.com/cgi/network/net_net.cgi?term=10&id=145097) | 155 | 2.8E-12 |
| Reproduction_Feeding and Neurohormone signaling | 210 | 7.45E-03 |
| [Neurophysiological process_Transmission of nerve impulse](http://portal.genego.com/cgi/network/net_net.cgi?term=10&id=145200) | 212 | 4.87E-02 |
| **Term - Diseases_UP** | **Count** | **P-Value** |
| Sensation | 58 | 1.66E-26 |
| [Nervous System Physiological Phenomena](http://portal.genego.com/cgi/entity_page.cgi?term=61&id=-1157372704) | 65 | 4.56E-25 |
| [Adrenocortical Hyperfunction](http://portal.genego.com/cgi/entity_page.cgi?term=61&id=-1401837744) | 79 | 1.02E-22 |
