# Supplementary material for: Gene Expression Signature Predictive of Neuroendocrine Transformation in Prostate Adenocarcinoma
Source: Int J Mol Sci. 2020 Feb 6;21(3):1078. doi: 10.3390/ijms21031078 (PMC7037893; doi:10.3390/ijms21031078)
Supplement: Supplementary file 1 [file ijms-21-01078-s001.zip › ijms-690854-supplementary-final/Supplementary_material/Supplementary Table 6.docx]

**Supplementary Table 6.** Ranking positions and enrichment p-values of AR and ESR1 among enriched transcription factors found for up- and down-regulated genes in three independent datasets of prostate cancer cell lines / organotypic slice cultures treated with estradiol or overexpressing ESR1 (FDR < 0.05).

|  | GSE43988 - VCaP cells overexpressing ESR1 | | GSE37531 - LNCaP cells treated with estradiol | | OSC11 treated with estradiol (E2) | |
| --- | --- | --- | --- | --- | --- | --- |
|  | **UP** | **DOWN** | **UP** | **DOWN** | **UP** | **DOWN** |
| AR | 1/122  (pv 2.06E-55) | 26/422  (pv 7.89E-54) | 20/115  (pv 3.38E-08) | 31/229  (pv 4.58E-09) | 1/195  (pv 1.17E-39) | 50/249  (pv 2.67E-06) |
| ESR1 | 14/122  (pv 4.74E-08) | 2/422  (pv 0) | 9/115  (pv 5.84E-12) | 33/229  (pv 1.9E-09) | 10/195  (pv 6.59E-22) | 13/249  (pv 1.88E-12) |
